# The complete chloroplast genome of *Fraxinus chiisanensis* (Oleaceae)

**DOI:** 10.1080/23802359.2017.1407684

**Published:** 2017-11-24

**Authors:** Sang-Chul Kim, Jei-Wan Lee, Seung-Hoon Baek, Min-Woo Lee, Kyung-Nak Hong

**Affiliations:** Division of Forest Genetic Resources, National Institute of Forest Science, Suwon, Republic of Korea

**Keywords:** *Fraxinus chiisanensis*, complete chloroplast genome, phylogenetic analysis, Oleaceae

## Abstract

*Fraxinus chiisanensis* is endemic to Korea and restricted to local habitats with an extremely limited population size. The chloroplast genomic information can be used to formulate a comprehensive conservation strategy. In this study, the complete chloroplast (cp) genome sequence of *F. chiisanensis* was determined using next-generation sequencing. The entire cp genome was determined to be 155,571 bp in length. It contained large single-copy (LSC) and small single-copy (SSC) regions of 86,412 of 17,767 bp, respectively, which were separated by a pair of 25,696 bp inverted repeat (IR) regions. The genome contained 132 genes, including 87 protein-coding genes, 37 tRNA genes, and eight rRNA genes. Among the 132 genes, seven coding genes, seven tRNA genes, and four rRNA genes occur in the two IR regions. The phylogenetic position of *F. chiisanensis* in Oleaceae was closely clustered with *Olea*, *Chionanthus retusus*, and *Hesperelaea palmeri* as sister species.

The genus *Fraxinus* L. comprises 43 species occurring in temperate and subtropical regions of the northern hemisphere (Wallander 2008). *Fraxinus chiisanensis* Nakai was previously known as a natural hybrid of *F. mandshurica* Rupr. between *F. rhynchophylla* Hance (Lee 1980). However, it was found to be included at sect. Melioides, which is distributed in North America in molecular phylogeny studies using ITS (Wallander 2008). *Fraxinus chiisanensis* is restricted to local habitats with an extremely limited population size: it is endemic to Korea and is mainly distributed in Chungcheongbuk-do, Jeollabuk-do, and Jeollanam-do provinces. It was included in The IUCN Red List of Threatened Species (http://www.iucnredlist.org/details/13188447/0). So, measures for conservation and restoration of this plant are urgently needed. It is necessary to develop genomic resources for *F. chiisanensis* to provide intragenic information for its conservation and valuable information about the course of evolution of *Fraxinus*.

Plant materials were sampled from the conservation area of the Forest Genetic Resources Department of the National Institute of Forest Science (in Suwon; N: 37° 15′ 8.74″, E: 126° 57′ 13.2″) and its genomic DNA was isolated from fresh leaves using a Plasmid SV mini kit (GeneAll, Seoul, Korea) and stored in a DNA bank in the Forest Genetic Resources Department (NIFS_0122059322). The whole genome sequencing was conducted on the Ion torrent Platform (Life Technologies, Carlsbad, CA). The filtered sequences were assembled with reference sequence of *Olea europaea* (GenBank: NC013707). The sequenced fragments were assembled using Geneious 10.2.3 (Biomatters, Auckland, New Zealand; Kearse et al. 2012). Annotation was performed using the DOGMA (http://dogma.ccbb.utexas.edu/) and BLAST searches. All the tRNA sequences were confirmed using the web-based online tool, tRNAScan-SE (Schattner et al. 2005) with default settings to corroborate tRNA boundaries identified by Geneious. The maximum likelihood (ML) tree searches and ML bootstrap searches were performed using the RAxML Blackbox web-server (http://phylobench.vital-it.ch/raxml-bb/, Stamatakis et al. 2008) using 79 protein-coding genes of the plastid genomes of nine species. The RAxML analyses were run with a rapid Bootstrap analysis using a random starting tree and 100 ML bootstrap replicates.

The plastome of *F. chiisanensis* was determined to comprise double stranded, circular DNA of 155,571 bp containing two inverted repeat (IR) regions of 25,696 bp each, separated by large single-copy (LSC) and small single-copy (SSC) regions of 86,412 and 17,767 bp, respectively (NCBI acc. no. KY859413). The genome contained 132 genes, including 87 protein-coding genes, 37 tRNA genes, and eight rRNA genes. The seven protein-coding genes, seven tRNA genes and four rRNA genes were duplicated in IR region. Fifteen genes contained one intron and two genes (*clpP* and *ycf3*) contained two introns. The *rps12* gene is a trans-spliced gene with two duplicated 3′-end exons in IR regions and one 5′ end exon in the LSC region. The ML phylogenetic tree constructed from the genomes of nine species (Figure 1) in Oleaceae (using the outgroup *Premna microphylla*) showed that *F. chiisanensis* was most closely related to *Olea*, *Chionanthus retusus*, and *Hesperelaea palmeri* as sister species as expected based on previous research (Wallander and Albert 2000).

**Figure 1.**
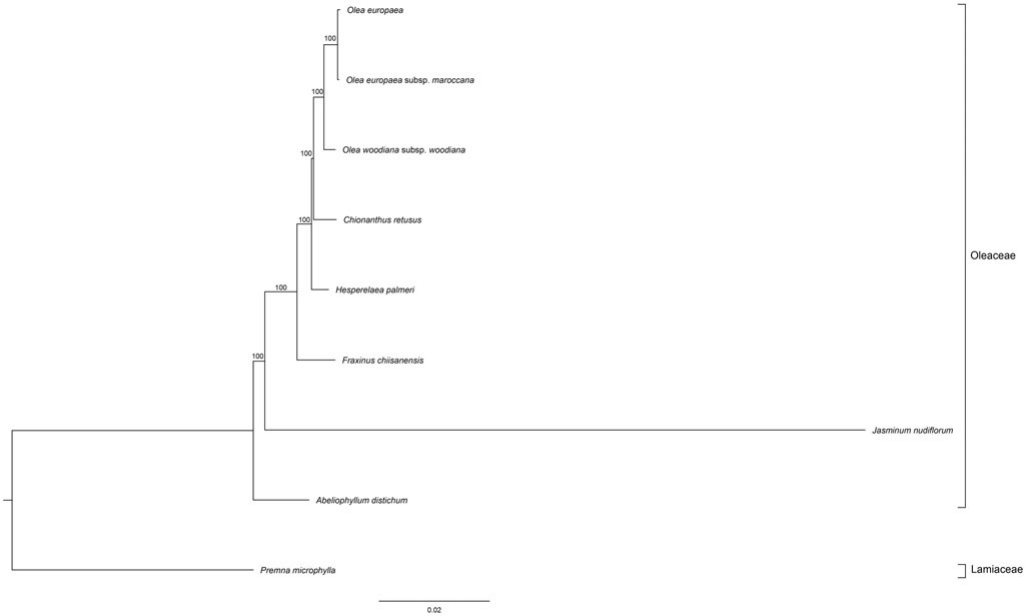
The phylogenetic tree based on the nine complete chloroplast genome sequences. Accession numbers: *Olea europaea* (NC013707), *Olea europaea* subsp. *maroccana* (NC015623), *Olea woodiana* subsp. *woodiana* (NC015608), *Chionanthus retusus* (NC035000), *Hesperelaea palmeri* (NC025787), *Jasminum nudiflorum* (NC008407), *Abeliophyllum distichum* (NC031445), and *Premna microphylla* (NC026291).
